# Enhancement of Anti-Proliferative Activity of the Extracts from Dehulled Adlay by Fermentation with *Bacillus* *subtilis*

**DOI:** 10.3390/foods10122959

**Published:** 2021-12-01

**Authors:** Anyan Wen, Yong Zhu, Muhammad Mazhar, Likang Qin, Haiying Zeng, Yi Zhu

**Affiliations:** 1 School of Liquor and Food Engineering, Guizhou University, Guiyang 550025, China; aywen@gzu.edu.cn (A.W.); zhuyonghappycool@163.com (Y.Z.); hyzeng1@gzu.edu.cn (H.Z.); 2 College of Life Sciences, Guizhou University, Guiyang 550025, China; mazharmicrobiologist@gmail.com; 3 Key Laboratory of Agricultural and Animal Products Storage and Processing of Guizhou Province, Guiyang 550025, China; 4 National & Local Joint Engineering Center for the Development and Utilization Technology of Drug and Food Resources in Southwest China, Guiyang 550025, China; 5 Plant Protection and Plant Quarantine Station of Guizhou Province, Guiyang 550001, China; Zy5286581@126.com

**Keywords:** dehulled adlay, *Bacillussubtilis* BJ3-2, anti-proliferative activity, bioactive components

## Abstract

Dehulled adlay was fermented with *Bacillus subtilis* BJ3-2, the anti-proliferative activities of the extracts from fermented dehulled adlay were investigated with six types of tumor cells, and then the bioactive components and the anti-proliferative mechanism were primarily explored. Results showed that all the extracts of *B.* *subtilis*-fermented dehulled adlay (BDA) and dehulled adlay (DA) had no inhibition effect on human embryonic kidney 239T cells. The anti-proliferative activities of the extracts from BDA against six types of tumor cells were almost always significantly higher than DA. Compared with others, the n-butanol extract of BDA (BDA-Nb) exhibited stronger anti-proliferative activities against human leukemia K562 cells and human non-small cell lung cancer A549 cells. Importantly, the anti-proliferative activity of fermented dehulled adlay against K562 cells was firstly discovered. Meanwhile, BDA-Nb was rich in tetramethylpyrazine, γ-aminobutyric acid, protocatechuic, 2,3,4-trihydroxybenzoic, chlorogenic, p-hydroxybenzoic, caffeic, trans-cinnamic, ferulic acids, and rutin. BDA-Nb induced the proliferative inhibition of K562 and A549 cells due to abnormal cell morphology, the increased cell population in G1 phase and apoptosis rate, the downregulation of Bcl-2, and the upregulation of Bax and caspase-3/8/9. These results indicate that dehulled adlay fermented with *B.* *subtilis* could be a potential therapeutic agent for leukemia and lung cancer.

## 1. Introduction

*Bacillussubtilis*, a Gram-positive bacterium, has a Generally Recognized as Safe status. *B.subtilis* has been widely utilized as an important starter culture for cereal due to its fast reproduction, easy culture, strong adversity, and no pathogenicity [1]. Recent research showed that the total phenolic content and antioxidant activities in oats were much increased after fermentation with *B.* *subtilis*, and β-glucosidase, α-amylase, and cellulase have an important impact on phenolic release [2]. The proteolysis and anti-oxidative properties of chickpeas were improved by solid-state fermentation with *B.* *subtilis*, and chickpea proteins were degraded to low-molecular-weight peptides during fermentation [3]. Meanwhile, Dai et al. [4] suggested that the nutritional value and antioxidant and antihypertensive activities in soybean meal were improved after fermentation by *B.* *subtilis*. Therefore, the development of cereal fermented by *B.* *subtilis* to improve health and nutritional benefits has attracted increasing attention.

Adlay (*Coix lacryma-jobi* L. var. *ma-yuen* Stapf) is a nourishing food and traditional Chinese medicine due to the bioactive phytochemicals, including polysaccharide, coixenolide, coixol, phenolics, lignans, steroids, and lactams [5]. The fractions, extracts, and isolated compounds from adlay exhibited multiple biological functions [6]. To obtain novel promising bioactive components and improve its functional effects, adlay is generally fermented with bacteria or fungi. Adlay fermented with *Saccharomyces cerevisiae* reduced the incidence of collagen-induced arthritis, and ferulic acid was isolated from fermented adlay extracts, suggesting that fermented adlay was a potential agent to alleviate anti-rheumatoid arthritis [7]. *Monascus purpureus*-fermented adlay, with high contents of γ-tocotrienol, γ-oryzanol, and coixenolide, exhibited higher antioxidant activity and anticancer activity against human laryngeal carcinoma cells HEp2 [8]. *Lactobacillus*-fermented adlay-soymilk showed anti-inflammatory effects in lipopolysaccharide-induced macrophages by suppressing the expression of NF-kappa B [9]. Interestingly, *Bacillus*-fermented adlay dramatically improved the antioxidant status, lipid metabolism, and intestinal microflora in hyperlipidemic hamsters, indicating that *B.* *subtilis*-fermented adlay could be used as a functional food for cardiovascular and intestinal health [10]. In previous studies, we also found that *B.* *subtilis*-fermented dehulled adlay (BDA) contained high levels of tetramethylpyrazine (TMP), phenolics, flavonoids, γ-aminobutyric acid (GABA), and triterpenoids, and exhibited strong antioxidant activity of alcoholic extract [11,12,13]. Therefore, it is necessary to further investigate whether the anti-proliferative activity of adlay is improved after fermentation by *B.* *subtilis*.

In this study, the anti-proliferative activities of different extracts from BDA were evaluated, and the bioactive components and possible mechanism of the effective extract were explored. Firstly, the anti-proliferative activities of different extracts were evaluated on six types of cancerous cell and human embryonic kidney 239T cells to confirm the effective extract. Secondly, the bioactive components of the effective extract were detected by high-performance liquid chromatography. Finally, the cell morphology, cell cycle, apoptosis rate, and the expression of apoptosis-related proteins were used to explore the possible mechanism of the effective extract on the anti-proliferative activity.

## 2. Materials and Methods

### 2.1. Materials and Chemicals

Dehulled adlay (DA) was obtained from Xingren, Guizhou Province, China (N 25°18′, E 105°34′) and sealed in plastic bags and stored until use (4 °C). *B.* *subtilis* BJ3-2 was provided by Dr. Wu (College of life sciences, Guizhou University, Guizhou, China). Human glioma U251 cells, human hepatocellular carcinoma HepG-2 cells, human colonic cancer Hct-15 cells, human non-small cell lung cancer A549 cells, human pancreatic cancer Panc-1cells, human leukemia K562 cells, and human embryonic kidney 239T cells were obtained from the Shanghai Institute of Cell Biology, Chinese Academy of Sciences (Shanghai, China). DMEM medium, RPMI-1640 medium, fetal bovine serum (FBS), and streptomycin were obtained from Gibco (Grand Island, NY, USA). Phosphate buffer solution (PBS), MTT reagent, dimethyl sulfoxide (DMSO), and Hoechst 33258 were purchased from Solarbio Science and Technology Ltd. (Beijing, China). Specific primary antibodies against Bax (ab32503), Bcl (ab32124), Caspase-3 (ab32351), Caspase-8 (ab32397), Caspase-9 (ab32539), and glyceraldehyde-3-phosphate dehydrogenase (GAPDH) (ab8245) were purchased from Abcam Technology, Inc. (Danvers, MA, USA). TMP, protocatechuic, 2,3,4-trihydroxybenzoic, caffeic, chlorogenic, p-hydroxybenzoic, trans-cinnamic and ferulic acids, and rutin were obtained from Sigma Chemical Co. (St. Louis, MO, USA).

### 2.2. Preparation of Different Extracts from Samples

*B. subtilis*-fermented dehulled adlay (BDA) was prepared according to the previous method by Wen et al. [13]. Approximately 30 g of dehulled adlay were washed and soaked overnight in distilled water. After draining, the soaked dehulled adlay was mixed with 42 mL of distilled water (*w/v*), and then steam-sterilized at 121 °C for 30 min. After cooling to room temperature, the steamed dehulled adlay was inoculated with *B.subtilis* BJ3-2, and then incubated at 7% and 40 °C for 72 h. After fermentation, BDA samples were subjected to freeze-vacuum drying, blended into a powder, sieved (60 mesh), sealed in plastic bags, and stored until use (4 °C). Then, the extracts of dried DBA powder were extracted according to previous methods with minor modifications [14,15,16]. The dried BDA powder (10 g) was refluxed with 80% ethanol (100 mL) in a water bath (80 °C, 2 h), and then filtered with filter paper. The filtrate was collected, and then the residue was re-extracted twice according to the same processing. The filtrate was then collected together and concentrated at 45 °C in a rotary evaporator to give crude ethanol extracts. For preliminary separation of BDA, the crude extract was fractionated using different solvents, and the solvents were petroleum ether (BDA-Pe), ethyl acetate (BDA-Ea), n-butanol (BDA-Nb), and aqueous (BDA-Aq) in sequence. All the extracts were concentrated, freeze-dried, and stored at 4 °C till further use. Similarly, four extracts from DA powder were extracted according to the same process and respectively named: DA-Pe, DA-Ea, DA-Nb, and DA-Aq.

### 2.3. Cell Culture

HepG-2, Hct-15, A549, and 239T cells were maintained in RPMI-1640 medium containing 1% streptomycin and 10% (*v*/*v*) heat-inactivated FBS. U251, Panc-1, and K562 cells were maintained in DMEM medium containing 1% streptomycin and 10% (*v*/*v*) heat-inactivated FBS. All cells were incubated at 37 °C in a humidified incubator containing 5% CO_2_.

### 2.4. Cell Viability Assay

The cell viability of the extracts from BDA and DA was evaluated with the MTT assay [17]. Briefly, 10^4^ cells were added in each well of the 96-well culture plate for 24 h in a humidified incubator containing 5% CO_2_ at 37 °C. Different concentrations of four extracts (25–1000 μg/mL) were added into each well. After 24 h of incubation, the medium was removed. Then, the cells were washed twice with PBS and 10 μL of MTT solution (5 mg/mL) were added in each well at 37 °C for 4 h. After the incubation, the MTT solution was discarded and 100 μL of DMSO were added into each well to dissolve formazan crystals under mechanical shaking for 10 min. The absorbance at 570 nm was measured with a universal microplate reader (ELx800, Bio-tek instruments Inc., Winooski, VT, USA).

### 2.5. Determination of Bioactive Compositions

TMP was detected with the high-performance liquid chromatography system (Agilent 1260) described by Chen et al. [18]. The chromatography was performed on a Phecda-C18 (250 mm × 4.6 mm, 5 μm) column at 45 °C, with an aqueous mobile phase (1% acetic acid and 0.05% trifluoroacetic acid in water, pH 2.5)–(methanol) (70:30, *v*/*v*). The flow rate was 0.8 mL/min and the UV detection wavelength was 297 nm. The injection volume of each sample was 20 μL.

GABA was determined according to the method described by Park et al. [19]. The derivative was quantitatively analyzed by HPLC with a Phecda-C18 (250 mm × 4.6 mm, 5 μm). The mobile phase was developed using 50% (*v*/*v*) acetonitrile (A) and phosphate butter (0.02 mol/L, pH 7.0) (B) at 1.0 mL/min. The gradient elution profile was as follows: 16–100% A at 0–20 min; 100–16% A at 20–26 min; 16% A at 26–30 min. The UV detection was set at a wavelength of 360 nm, and the injection volume of each sample was 20 μL.

Total phenoics, total flavonoids, and phenolic compounds were analyzed according to the method described by Xu et al. with minor modifications [20]. First, 0.4 mL of properly diluted extracts, 2.6 mL of deionized water, and 0.5 mL of Folin–Ciocalteu reagent were transferred to a 10 mL brown volumetric flask. After 6 min, 1.5 mL of 20% sodium carbonate solution (*w/v*) were added, and distilled water was used to bring up the mixture to 10 mL. After 2 h of incubation at 40 °C, the absorbance was measured at 760 nm using a spectrophotometer (Metash V-1800) and the content of total phenolics was expressed as gallic acid equivalent (mg GAE/g DW). A 500 μL aliquot of the extract was added to 2 mL of 30% ethanol, and then 150 μL of 5% NaNO_2_ solution were added and incubated for 6 min. Subsequently, 150 μL of 10% AlCl_3_·6H_2_O solution were added, and the mixture was incubated for 6 min before adding 2 mL of 1 mol/L NaOH solution. After the volume of the mixture was brought up to 5 mL with distilled water, the absorbance was measured 15 min later at 510 nm, and the content of total flavonoids was expressed as rutin equivalent (mg RE/g DW). Elution solvents used for phenolic compounds were acetonitrile (A) and 0.1% (*v*/*v*) formic acid (B). The gradient elution procedure was performed according to the following program: 0–5 min, 5–15% A; 5–35 min, 15–35% A; 35–40 min, 35–45% A; 40–50 min, 45–5% A. The column temperature, flow rate, and UV detection wavelength were respectively set as 30 °C, 1 mL/min, and 280 nm.

### 2.6. Morphological Observations

The morphological changes were analyzed according to the method by Rajan et al. [21]. K562 and A549 cells (5 × 10^4^) were seeded into 6-well plates and then different concentrations of BDA-Nb (50, 100, 200 μg/mL) were added for the 24  h reaction. Then, the cells were harvested, washed twice with PBS, and then stained for 10 min with Hoechst 33258. The stained nuclei were observed under a Nikon TE2000-U fluorescence microscope (Nikon instruments Inc., Tokyo, Japan).

### 2.7. Flow Cytometry Analysis

K562 and A549 cells were cultivated for 20 h in 6-well plates (5 × 10^5^/well). Then, the cells were subjected to 50–200 μg/mL BDA-Nb for 24 h for the subsequent analysis of cell cycle. The cells were harvested, washed with PBS, and centrifuged (2000 rpm, 5 min). After the supernatant was removed, the cells were suspended in 70% ethanol overnight (4 °C). Then, the cells were washed with ice-cold PBS and centrifuged (1000 rpm, 3 min). Cell pellets were then suspended in 500 μL of staining solution containing 10% Rnase A and 90% propidium iodide solution and incubated for 30 min at 37 °C. The samples were examined by a flow cytometer.

The cell apoptosis was assessed with an Annexin V-EGFP Apoptosis Detection Kit. The cells were treated with 50–200 μg/mL BDA-Nb for 24 h, harvested, washed in PBS, and centrifuged (2000 rpm, 5 min). After the supernatant was removed, the cells were suspended in 500 μL of binding buffer and stained at room temperature for 15 min in 5 μL of Annexin V-FITC and 5 μL of propidium iodide in a dark place. The samples were finally analyzed with a flow cytometer.

### 2.8. Western Blot Analysis

K562 and A549 cells were treated for 24 h with 50–200 μg/mL BDA-Nb, washed twice with PBS, and lysed in RIPA Buffer. Proteins were separated on 10% SDS-polyacrylamide gels and then transferred to a PVDF membrane. The obtained membranes were blocked for 1 h with 5% free-fat milk in TBST buffer and then incubated with primary antibodies (GAPDH, Bcl-2, Bax, Caspase-3, Caspase-8, and Caspase-9) overnight at 4 °C. After the treated membranes were washed with TBST three times, they were incubated for 2 h at 4 °C with anti-rabbit IgG-alkaline peroxidase-conjugated secondary antibody and then washed twice with PBS. Finally, the membranes were detected with a ChemiDocTM Imaging System (Bio-Rad Laboratories, Inc., Hercules, CA, USA).

### 2.9. Statistical Analysis

All the samples were detected in triplicate and the data were expressed as mean ± SD. The statistical analysis was carried out in SPSS 17.0 software (SPSS Inc., Chicago, IL, USA). The differences among the results were analyzed by Duncan’s test and analysis of variance (ANOVA).

## 3. Results

### 3.1. Fermentation Improved the Anti-Proliferative Activity of Dehulled Adlay

**Figure 1:**
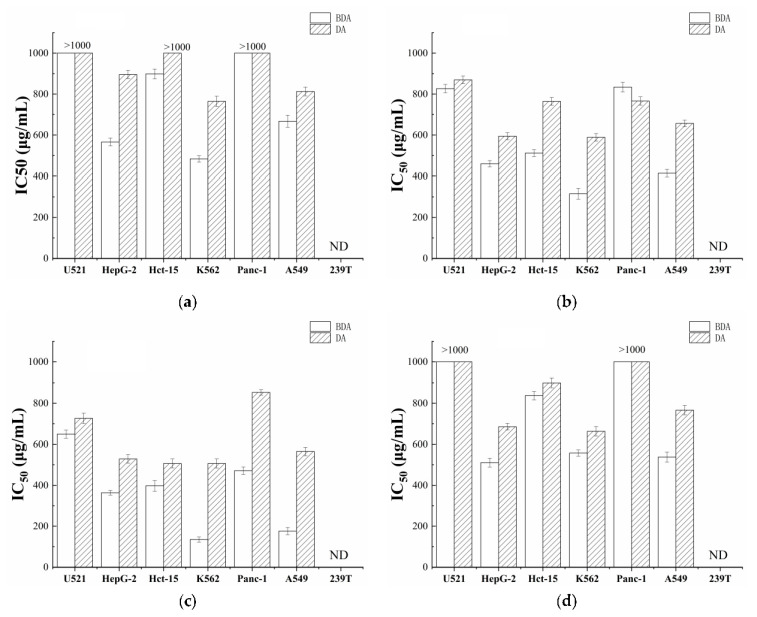
The IC_50_ values of petroleum ether (**a**), ethyl acetate (**b**), n-butanol (**c**), and aqueous (**d**) extracts from BDA and DA, respectively. All data values are expressed as the mean ± SD (n = 3). ND, no detect.

To compare the anti-proliferative effects of BDA and DA, all cells were exposed to different concentrations (25–1000 μg/mL) of the extracts, respectively. As shown in Figure 1, all the extracts from BDA and DA had no inhibiting effect on human embryonic kidney 239T cells. The IC_50_ values of the extracts from BDA were almost always significantly lower than that of DA, suggesting the extracts of BDA showed stronger cytotoxic activity than that of DA. Furthermore, the cytotoxic activity data of the four extracts from BDA on the proliferation of six human tumor cell lines suggested the higher inhibition effects on all tumor cells in BDA-Nb (Figure 1c), especially K562 and A549. The IC_50_ values of BDA-Nb on K562 and A549 were 134.55 and 176.11 μg/mL, respectively. Therefore, the bioactive components of BDA-Nb need further investigation.

### 3.2. Fermentation Increased the Bioactive Component Content of Dehulled Adlay

In order to compare the bioactive components of BDA-Nb and DA-Nb, the bioactive components were investigated with HPLC. As shown in Table 1, the total flavonoids contents of BDA-Nb and DA-Nb were 36.49 and 28.46 mg GAE/g, and the total phenolics contents were 45.60 and 32.16 mg RE/g, respectively. The TMP content of BDA-Nb was 4.62 mg/g dry basis, but TMP was not detected in DA-Nb. The GABA content of BDA-Nb was 13.90 mg/g dry basis, which was 2.63 times higher than of DA-Nb. Nine phenolic compounds (trans-cinnamic, protocatechuic, 2,3,4-trihydroxybenzoic, chlorogenic, p-hydroxybenzoic, ferulic, pcoumaric, caffeic acids, and rutin) were detected in DBA-Nb, and the components with the highest and lowest contents of phenolic compounds were respectively 2,3,4-trihydroxybenzoic acid (2.08 mg/g dry basis) and pcoumaric acid (0.21 mg/g dry basis). Except pcoumaric acid, the contents of phenolic compounds in DBA-Nb were higher than that of DA-Nb. The data indicated that the levels of total phenolics, total flavonoids, TMP, GABA, and phenolic compounds significantly increased in fermented dehulled adlay. Next, the treatment of K562 and A549 with BDA-Nb was further investigated. Additionally, K562 and A549 cell lines were treated with 50, 100, and 200 μg/mL BDA-Nb for 24 h; TMP and cisplatin were used as the positive control; and DMSO was used as a negative control.

### 3.3. BDA-Nb Inhibited the Growth of K562 and A549 Cells

**Figure 2:**
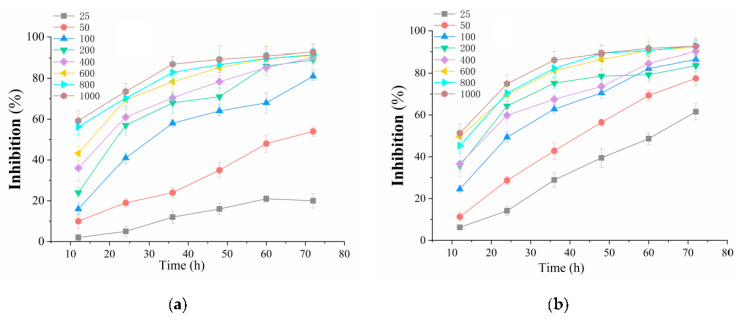
Effect of BDA-Nb on the viability of K562 (**a**) and A549 (**b**) cells at various concentrations for different time periods. All data values are expressed as the mean ± SD (n = 3).

To investigate the effects of BDA-Nb with different concentrations on the viability against K562 and A549 cells, MTT assays were performed to evaluate the proliferation of BDA-Nb in a time- and concentration-dependent manner. The treatment with BDA-Nb inhibited the growth of K562 and A549cells in a time- and concentration-dependent manner (shown in Figure 2). The results indicated that the cell survival rate gradually decreased with the increase in the BDA-Nb concentration and treatment time.

### 3.4. BDA-Nb Induced Morphological Changes in K562 and A549 Cells

**Figure 3:**
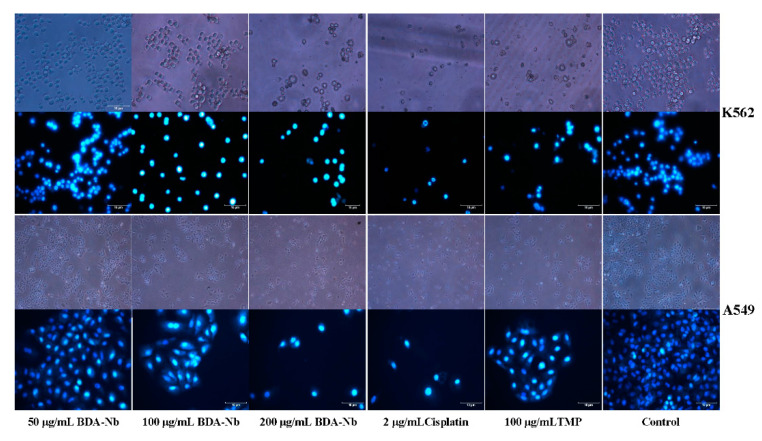
The cell morphology change of K562 and A549 cells treated with BDA-Nb of various concentrations.

To observe the morphological changes of K562 and A549 cells after treatment with BDA-Nb, the cells were stained with Hoechst 33258 and then examined under a fluorescence microscope. Figure 3 showed the untreated K562 cells exhibited the normal and regular cell shape and cell nuclear membrane integrity. K562 cells treated with BDA-Nb, TMP, and cisplatin displayed drastic morphological changes. The total number of cells was significantly decreased and accompanied by undesirable properties, including shrunken cells, a collapsed cytoskeleton, and vacuolar degeneration. The untreated A549 cells displayed an intact cell membrane, a polygon and tight arrangement, and adhered to walls. After the treatment with BDA-Nb, TMP, and cisplatin, A549 cells fell off and floated and a number of fragments were found. After staining with Hoechst 33258, the nucleus of untreated K562 and A549 cells displayed soft, faint, and homogeneous blue fluorescence. After treatment with BDA-Nb, TMP, and cisplatin, K562 and A549 cells showed a nuclear chromatin concentration, condensation, and margination with hyperchromatic and crowded nuclei. Thus, blocky-granular strong fluorescence with a dense concentration was observed and apoptotic bodies were detected, indicating that the abnormal morphological changes of K562 and A549 cells appeared after the treatment with BDA-Nb.

### 3.5. BDA-Nb Disturbed Cell Cycle Progression in K562 and A549 Cells

**Figure 4:**
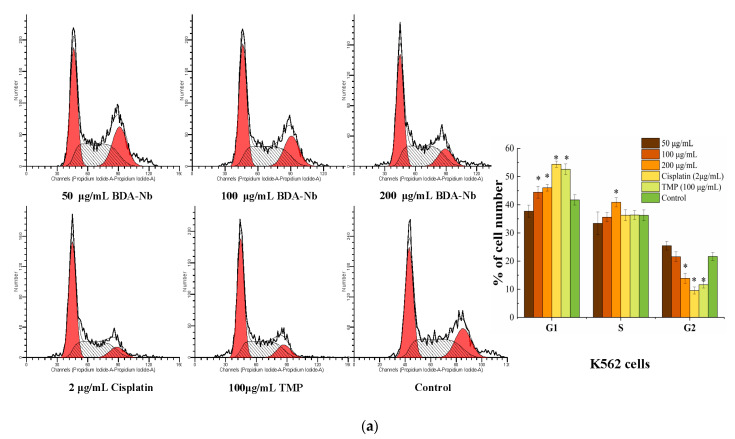

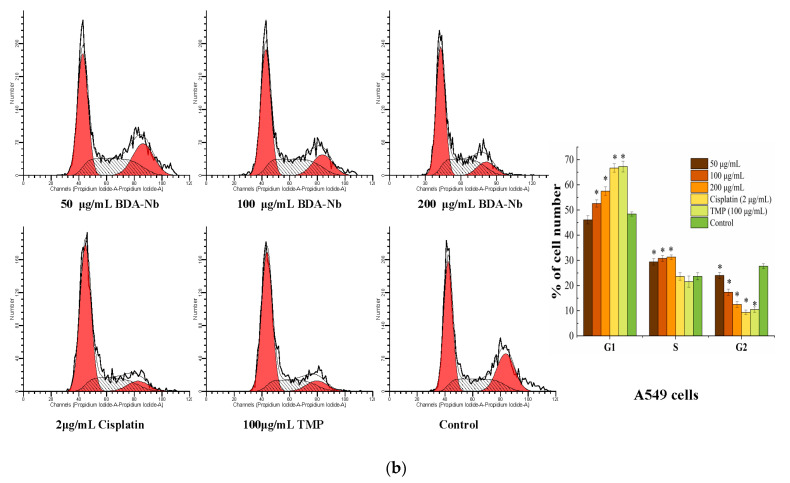
Flow cytometric analysis of BDA-Nb-induced cell cycle arrest in K562 (**a**) and A549 (**b**) cells. All data values are expressed as the mean ± SD (n = 3). * *p* < 0.05 versus control cells, which were not exposed to BDA-Nb.

To evaluate the effect of BDA-Nb on the cell cycle progression of K562 and A549 cells, the cell cycle profile of K562 and A549 cells treated with different concentrations of BDA-Nb was analyzed with a flow cytometer. The treatment with increasing concentrations of BDA-Nb had a dose–concentration effect on the cell cycle progression (Figure 4), suggesting a statistically significant increase in the accumulation of cells in the G1 phase (50 to 200 μg/mL BDA-Nb). Simultaneously, the accumulated cells in the G2 phase decreased after the treatment with BDA-Nb. After K562 cells were respectively treated with 50, 100, and 200 μg/mL BDA-Nb, the percentages of K562 cells in the G1 phase were 37.71%, 44.44%, and 46.03%, and the percentages of the G2 phase were 25.41%, 21.54%, and 13.89%, respectively. After A549 cells were respectively treated with 50, 100, and 200 μg/mL BDA-Nb, the percentages of A549 cells in the G1 phase were 46.09%, 52.57%, and 57.49%, and the percentages of A549 cells in the G2 phase were 24.03%, 17.14%, and 12.50%, respectively. However, the effects of BDA-Nb on cell cycle progression of K562 and A549 cells were weaker than the positive controls (cisplatin and TMP).

### 3.6. BDA-Nb Induced Apoptosis in K562 and A549 Cells

**Figure 5:**
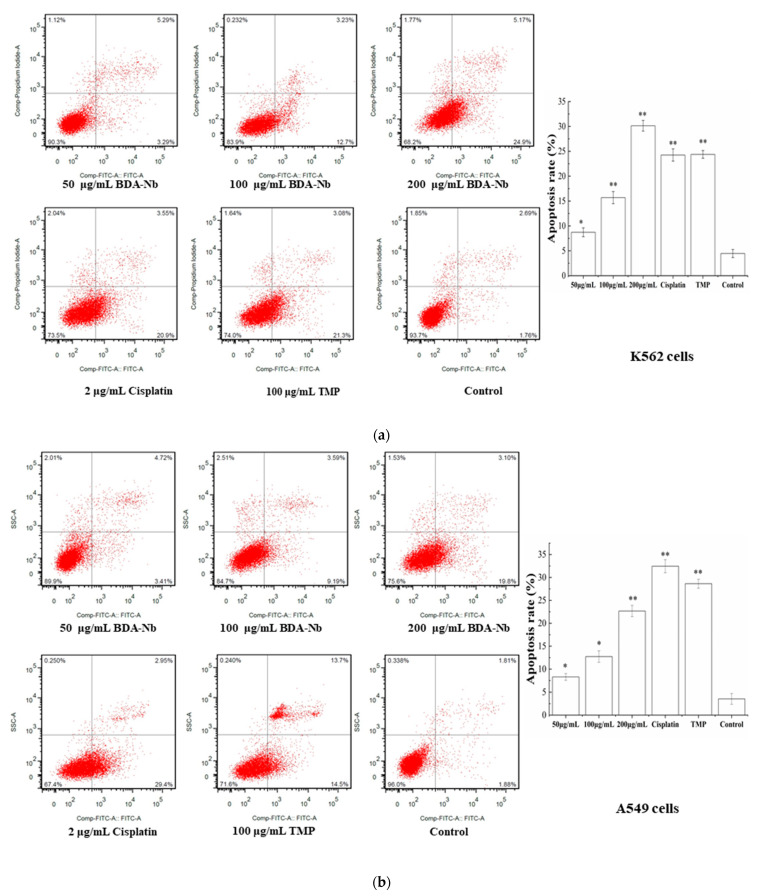
Flow cytometric analysis of BDA-Nb-induced apoptosis in K562 (**a**) and A549 (**b**) cells. All data values are expressed as the mean ± SD (n = 3). * *p* < 0.05 and ** *p* < 0.01versus control cells, which were not exposed to BDA-Nb.

To investigate the effect of BDA-Nb on apoptosis of K562 and A549 cells, cells treated BDA-Nb were stained with Annexin V/PI and then detected with a flow cytometer. Obvious apoptosis was observed in K562 and A549 cells treated with BDA-Nb in a dose-dependent manner, and the most obvious change was an increase in early apoptotic cells (Figure 5). As shown in Figure 4a, after the treatment with 50, 100, and 200 μg/mL BDA-Nb, the apoptotic percentages of K562 cells were respectively 8.73%, 15.70%, and 30.14%, and the apoptotic percentage of cells in the control was 4.41%. The apoptotic percentage of K562 in the high-dose group (200 μg/mL) was higher than that of the cisplatin (24.26%) and TMP (24.39%) groups. After the treatment with 50, 100, and 200 μg/mL BDA-Nb, the apoptotic percentages in A549 cells were 8.29%, 12.73%, and 22.67%, respectively (Figure 5b).

### 3.7. BDA-Nb Induced Protein Expression in K562 and A549 Cells

**Figure 6:**
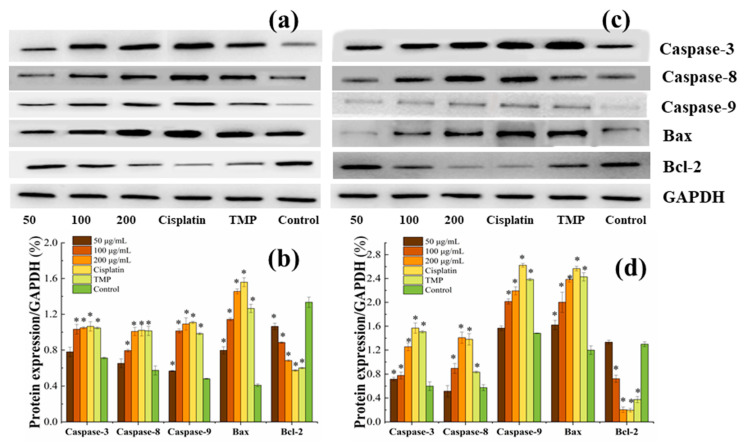
Effect of BDA-Nb on apoptosis-related protein expression in K562 (**a**,**b**) and A549 (**c**,**d**) cells. All data values are expressed as the mean ± SD (n = 3). * *p* < 0.05 versus control cells, which were not exposed to BDA-Nb.

To explore the effect of BDA-Nb on the protein expression in K562 and A549 cells, key proteins related to mitochondria-mediated intrinsic apoptosis, such as Bcl-2, Bax, caspase-3, caspase-8, and caspase-9, were evaluated. With the increase in the concentration of BDA-Nb, the expression of Bcl-2 decreased, whereas the expression of caspase-9, caspase-8, caspase-3, and Bax was significantly increased (Figure 6). Therefore, our findings revealed that BDA-Nb exhibited significant effects on protein expression of K562 and A549 cells.

## 4. Discussion

In this study, dehulled adlay fermented by *B.* *subtilis* had stronger cytotoxic activity compared with dehulled adlay (Figure 1), indicating that the anti-proliferative activity of dehulled adlay was obviously improved after fermentation. Wang et al. [11] also showed that *Bacillus*-fermented adlay significantly reduced serum and liver cholesterol concentrations, raised serum antioxidant activity, and balanced microbial populations. Tseng et al. [22] found that methanolic extracts from *Monascal* adlay showed higher antioxidant activity than uninoculated adlay products. Similarly, solid-state wheat, quinoa, and lupin fermented with *Lactobacillus reuteri* K777 and *Lb. plantarum* K779 had the stronger antioxidant ability and anti-proliferative activities in MCF-7 and Caco-2 cell lines than unfermented products [23]. We postulated that the higher anti-proliferative activity of BDA may be primarily attributed to: (1) bioactive components released during fermentation and (2) new effective components generated by way of biotransformation during bacterial metabolism. These results suggested that the anti-proliferative activity of dehulled adlay was improved through fermentation.

Compared with the other three extracts of fermented dehulled adlay, BDA-Nb exhibited the higher proliferative inhibition effects against six human tumor cell lines, especially K562 and A549 (IC_50_ values of 134.55 and 176.11 μg/mL, respectively) as shown in Figure 1. Similarly, Chen et al. [24] observed that that n-butanol extract from defatted adlay seed meal had higher anti-proliferative activities against MCF-7, HepG2, and Caco-2 cells than aqueous and acetone extracts. Thus, the bioactive components from adlay were effectively extracted by n-butanol. Furthermore, previous studies demonstrated a polysaccharide fraction, ethyl acetate-soluble fraction, and Kanglaite injection extracted from adlay exhibited strong anti-proliferative effects on A549 [25,26,27], but we found that the extract from adlay was capable of inhibiting the proliferation in K562 cells for the first time.

The bioactive components of dehulled adlay were improved after fermentation with *B.* *subtilis*. BDA-Nb contained high contents of TMP and GABA, and appreciable concentrations of protocatechuic, 2,3,4-trihydroxybenzoic, chlorogenic, p-hydroxybenzoic, caffeic, trans-cinnamic, ferulic acids, and rutin (Table 1). Interestingly, the contents of TMP, GABA, protocatechuic, 2,3,4-trihydroxybenzoic, caffeic, trans-cinnamic, ferulic acids, and rutin in dehulled adlay were significantly increased after fermentation by *B. subtilis* BJ3-2. At present, the bioactive components in BDA proved to have anti-proliferative activity. Jia et al. [28] found that TMP inhibited angiogenesis and tumor growth of A549 cells by blocking the BMP/Smad/Id-1 signaling. Jia et al. [29] showed that small interfering RNA (siRNA) silencing Apollon gene combined with TMP significantly increased the proliferation inhibition rate and apoptosis rate of K562 cells. GABA inhibited the proliferation of colon cancer cells, pancreatic cancer cells, and cholangiocarcinoma cells QBC939 [30,31,32]. In fact, Chen et al. [24] isolated p-coumaric acid, ferulic acid, and rutin from n-butanol fraction of defatted adlay seed meal, and reported that these compounds exhibited anticancer activities in MCF-7, HepG-2, and CaCo-2 cancer cells. Li et al. [33] detected ferulic, p-hydroxybenzoic, caffeic acids, and rutin in free phenolic extract from tartary buckwheat bran with HPLC, and showed the anticancer activities of free phenolic extracts against human breast cancer MDA-MB-231 cells via the p38/MAP kinase pathway. Chai et al. [34] indicated that aqueous extracts from Phymatopteris triloba, containing high contents of protocatechuic and p-hydroxybenzoic acids, produced about 30% anti-proliferative activity on K562 cells (500 μg dry matter/mL). Therefore, the bioactive compounds of adlay fermented by *B.* *subtilis* were improved and had stronger anti-proliferative activity. It is necessary to further investigate the possible mechanism of anti-proliferative activities of BDA-Nb through many indexes, including cell morphology, cell cycle, apoptosis, and expression of apoptosis-related proteins in K562 and A549 cells.

Firstly, BDA-Nb obviously decreased the proliferating activity of K562 and A549 cells in a concentration- and time-dependent manner (Figure 2). With the increase in the treatment time and concentration of BDA-Nb, the proliferation inhibition level increased. Son et al. [35] also observed that the cell proliferation inhibition in the cells treated with Coix lacryma-jobi sprout extract increased in a concentration- and time-dependent manner. Meanwhile, K562 and A549 cells showed abnormal morphological features, including low growth density, nuclear chromatin concentration, condensation, and margination (Figure 3). Apoptosis was a programmed cell death process with the changes of chromosomal DNA fragmentation, cellular blebbing, cell contraction, chromatinorrhexis, and chromatin condensation [36].

Next, BDA-Nb led to significant accumulation of K562 and A549 cells in the G1 phase, reduced cells in the G2 phase, and induced early apoptosis with a concentration-dependent manner (Figure 4), thus resulting in an overall delay in the cell cycle and a decrease in cell proliferation. Previous studies certainly confirmed that increasing the cell population in the G1 phase and decreasing the cell population in the G2 phase were the reasons for the proliferation inhibition of cancer cells [37]. TMP treatment of human bladder cancer Pumc-91 cells resulted in an increased percentage of G1 phase cells and decreased percentage of S phase cells [38]. Similarly, phenolic-rich extracts decreased the percentage of cells in the G1 phase and caused cell cycle arrest in the S phase [39]. Apoptosis assays confirmed that BDA-Nb could promote cell apoptosis, and the most striking change was a large number of apoptotic cells detected in the early stage (Figure 5). Lu et al. [25] reported that a polysaccharide fraction of adlay induced early apoptosis of A549 cells. Therefore, we could draw the conclusion that promoting cell apoptosis and cell-division arrest were the reasons for the inhibition effect of BDA-Nb on the proliferation of K562 and A549 cells.

Furthermore, the expression of the anti-apoptotic proteins Bcl-2 was significantly downregulated, and the expressions of the proapoptotic protein Bax, caspase-3, caspase-8, and caspase-9 were upregulated after the treatment of BDA-Nb (Figure 6). The anti-apoptotic protein Bcl-2 could stabilize the mitochondrial membrane integrity by hampering caspase activation, Bax reallocation to the mitochondria, and apoptosis [40]. Bax, as one of the pro-apoptotic Bcl-2 family members, plays an important role in the release of cytochrome c from mitochondria, thus resulting in cell apoptosis. The apoptotic process of tumor cells could be activated by decreasing in Bcl-2 and increasing in Bax [37]. The activation of caspase-3 could induce cell apoptosis through inactivation of vital cellular proteins and nucleases, which triggered DNA fragmentation [41]. The sequential activation of caspase-3 played a vital role in the execution phase of cell apoptosis, and caspase-3 could interact with caspase-8 and caspase-9 [42]. Obviously, BDA-Nb had effects on the expression of apoptosis-related proteins in A549 and K562 cells and directly or indirectly induced apoptosis against K562 and A549 cells.

Additionally, the TMP concentration in the high-dose group of BDA-Nb was far lower than that in the TMP positive control, but the anti-proliferative activities of BDA-Nb on K562 and A549 cells were about the same as those in the TMP positive control. Thus, we inferred the synergistic or additive effects of TMP, 2,3,4-trihydroxybenzoic, protocatechuic, chlorogenic, p-hydroxybenzoic, ferulic, trans-cinnamic, caffeic acids, and rutin contributed to the strong antitumor abilities of BDA-Nb. Sasipawan et al. [43] found that the extract mixture of P. evecta exhibited higher cytotoxicity and more significant apoptosis induction than single hexane extracts in HepG2 cells. The ATR/FT-IR spectra revealed the presence of polyphenolic constituents, which had a synergistic effect on HepG2 cells. Dai et al. [44] also observed that anthocyanin and non-anthocyanin phenolics from blackberry extracts had synergistic or additive anticancer effects on HL-60 cells. Therefore, it is certainly worth further investigating the synergistic or additive effects of the bioactive components from BDA-Nb.

## 5. Conclusions

The extracts of BDA exhibited stronger anti-proliferative activities than that of DA, indicating that the anti-proliferative activity of dehulled adlay was enhanced by fermentation using *B.* *subtilis* BJ3-2. Meanwhile, BDA-Nb displayed the highest anti-proliferative potency against K562 and A549 cells. Furthermore, BDA-Nb treatment of K562 and A549 led to characteristic nuclei fragmentation, increased cell population in the G1 phase, and induced an increased apoptosis rate. Additionally, Western blotting clearly indicated that the expression of caspase-3, caspase-8, caspase-9, and Bax significantly increased, whereas the expression of Bcl-2 decreased in K562 and A549 cells. Notably, BDA-Nb contained high contents of TMP, GABA, 2,3,4-trihydroxybenzoic, protocatechuic, chlorogenic, p-hydroxybenzoic, ferulic, trans-cinnamic, caffeic acids, and rutin, which might have synergistic or additive anti-proliferative effects. Based on these results, we conclude that BDA-Nb is a potential therapeutic agent for the treatment of human chronic myelogenous leukemia and lung cancer. Exploration of the anti-cancer potential of BDA-Nb in in vivo animal models of cancer is still required.

## Figures and Tables

**Table 1:** The contents of bioactive components from BDA-Nb and DA-Nb (mg/g dry basis).

Bioactive Components	BDA-Nb	DA-Nb
Total phenolics (mg GAE/g dry basis)	36.49 ± 1.37 a	28.46 ± 1.67 b
Total flavonoids (mg RE/g dry basis)	45.60 ± 2.09 a	32.16 ± 1.87 b
TMP	4.62 ± 0.25 a	ND
GABA	13.90 ± 1.05 a	5.29 ± 1.16 b
Protocatechuic acid	1.11 ± 0.09 a	0.51 ± 0.08 b
2,3,4-Trihydroxybenzoic acid	2.08 ± 0.07 a	0.67 ± 0.12 b
Chlorogenic acids	0.45 ± 0.12 a	0.31 ± 0.07 a
p-Hydroxybenzoic acid	0.32 ± 0.05 a	0.22 ± 0.08 a
Caffeic acid	0.84 ± 0.08 a	0.24 ± 0.06 b
Trans-Cinnamic acid	0.95 ± 0.11 a	0.35 ± 0.07 b
Pcoumaric acid	0.21 ± 0.07 b	0.35 ± 0.05 a
Rutin	0.56 ± 0.06 a	0.34 ± 0.09 b
Ferulic Acid	0.63 ± 0.07 a	0.31 ± 0.13 b

Note: ND, no detect. Data are expressed as the mean ± SD (n = 3). Different lower-case letters in the same line indicate significant differences at *p* < 0.05. TMP: tetramethylpyrazine; GABA: γ-aminobutyric acid.

## Data Availability

Data is contained within the article.
